# Reliability and Validity of the Dutch Physical Activity Questionnaires for Children (PAQ-C) and Adolescents (PAQ-A)

**DOI:** 10.1186/2049-3258-72-47

**Published:** 2014-12-24

**Authors:** Liene Bervoets, Caroline Van Noten, Sofie Van Roosbroeck, Dominique Hansen, Kim Van Hoorenbeeck, Els Verheyen, Guido Van Hal, Vanessa Vankerckhoven

**Affiliations:** Faculty of Medicine and Life Sciences, Hasselt University, Martelarenlaan 42, 3500 Hasselt, Belgium; Faculty of Medicine and Health Sciences, University of Antwerp, Universiteitsplein 1, 2610 Wilrijk, Belgium; Department of Epidemiology and Social Medicine, University of Antwerp, Universiteitsplein 1, 2610 Wilrijk, Belgium; Heart Centre Hasselt, Jessa Hospital, Stadsomvaart 11, 3500 Hasselt, Belgium; Department of Paediatrics, Antwerp University Hospital, Wilrijkstraat 10, 2650 Edegem, Belgium; Vaccine & Infectious Disease Institute, University of Antwerp, Universiteitsplein 1, 2610 Wilrijk, Belgium

**Keywords:** Pediatrics, Physical activity, Reliability, Validation

## Abstract

**Background:**

This study was designed to validate the Dutch Physical Activity Questionnaires for Children (PAQ-C) and Adolescents (PAQ-A).

**Methods:**

After adjustment of the original Canadian PAQ-C and PAQ-A (i.e. translation/back-translation and evaluation by expert committee), content validity of both PAQs was assessed and calculated using item-level (I-CVI) and scale-level (S-CVI) content validity indexes. Inter-item and inter-rater reliability of 196 PAQ-C and 95 PAQ-A filled in by both children or adolescents and their parent, were evaluated. Inter-item reliability was calculated by Cronbach’s alpha (α) and inter-rater reliability was examined by percent observed agreement and weighted kappa (κ). Concurrent validity of PAQ-A was examined in a subsample of 28 obese and 16 normal-weight children by comparing it with concurrently measured physical activity using a maximal cardiopulmonary exercise test for the assessment of peak oxygen uptake (VO_2_ peak).

**Results:**

For both PAQs, I-CVI ranged 0.67-1.00. S-CVI was 0.89 for PAQ-C and 0.90 for PAQ-A. A total of 192 PAQ-C and 94 PAQ-A were fully completed by both child and parent. Cronbach’s α was 0.777 for PAQ-C and 0.758 for PAQ-A. Percent agreement ranged 59.9-74.0% for PAQ-C and 51.1-77.7% for PAQ-A, and weighted κ ranged 0.48-0.69 for PAQ-C and 0.51-0.68 for PAQ-A. The correlation between total PAQ-A score and VO_2_ peak – corrected for age, gender, height and weight – was 0.516 (p = 0.001).

**Conclusions:**

Both PAQs have an excellent content validity, an acceptable inter-item reliability and a moderate to good strength of inter-rater agreement. In addition, total PAQ-A score showed a moderate positive correlation with VO_2_ peak. Both PAQs have an acceptable to good reliability and validity, however, further validity testing is recommended to provide a more complete assessment of both PAQs.

**Electronic supplementary material:**

The online version of this article (doi:10.1186/2049-3258-72-47) contains supplementary material, which is available to authorized users.

## Background

Physical inactivity in children and adolescents has become a major issue in public health [1]. In Belgium, the Health Behavior in School-aged Children (HBSC) study showed that in 2010 only 10 to 30% of children aged 11, 13 and 15 were moderate to vigorous physically active during one hour per day [2]. Increasing physical activity is a key element in the treatment of several diseases, including childhood obesity and associated health conditions. Assessment of physical activity is therefore a topic of strong interest in public health research.

Various objective and subjective methods have already been suggested to evaluate the level of physical activity in children and adolescents [3]. Unfortunately, some objective measurements such as heart rate monitoring and accelerometry require special equipment and are more difficult to perform in children, especially on a large scale, compared to subjective measurements. Subjective methods to estimate physical activity including questionnaires, interviews and diaries, are preferred in large epidemiological studies.

The self-report Canadian PAQ-C and PAQ-A, developed by Kowalski et al. [4] are valid, cost-effective and feasible tools to evaluate physical activity in youth. Indeed, these questionnaires have been used to test for multiple psychometric properties, i.e. item and scale, test-retest reliability, internal consistency, sensitivity to gender and age differences, convergent validity, and construct validity [4–8]. All of these properties have been reported as acceptable to good.

In Flanders (the Dutch speaking part of Belgium), however, the reliability and validity of these questionnaires has not yet been evaluated in children and adolescents. Therefore, the principal aim of this study is to assess the reliability and validity of a Dutch version of the PAQ-C and PAQ-A in children and adolescents.

## Methods

### Ethical approval

The study was conducted in accordance with the ethical rules of the Helsinki Declaration. Informed consent was obtained from all children and their parents or legal guardian. The study protocol was approved by the Antwerp University Hospital Ethics Committee (Comité voor Medische Ethiek, Universitair Ziekenhuis Antwerpen, Wilrijkstraat 10, approval number 7/41/226).

### Content validity

Both PAQs were evaluated by an expert committee consisting of three professionals in Pediatrics, one professional in Sports Medicine, and two professionals in Epidemiology and Sociology (see Additional file 1 for Dutch PAQ-C and Additional file 2 for Dutch PAQ-A). At first, the original Canadian PAQs developed by Kowalski et al. [4] were translated into Dutch and then back-translated into English according to the ‘translation/back-translation’ method [9] by two bilinguals belonging to the expert committee. Next, the first question was adjusted according to the socio-cultural conditions and available sport activities in Flanders (Belgium): i.e. some sport activities from the original PAQs (rowing/canoeing, aerobics, street hockey, cross-country skiing, ice-hockey/ringette) were substituted by sport activities practiced in Flanders (tennis, athletics, combat sports, horse riding and gymnastics). Item-level (I-CVI) and scale-level (S-CVI) content validity indexes were calculated to measure content validity [10].

### Inter-item and inter-rater reliability

To assess the inter-item and inter-rater reliability of PAQ-C and PAQ-A, six primary and four secondary schools were selected at random from schools located in Antwerp and Limburg (Flanders, Belgium). The selection was based on the response rate of schools which were located in the vicinity of the university and/or home of the researchers involved. Children or adolescents and their parents were informed about this study by means of distribution of an information letter. Afterwards, all responders received a letter with detailed information on the study protocol and the appropriate physical activity questionnaires to be completed and returned. Parent–child reliability (i.e. inter-rater agreement) was evaluated based on the PAQs filled in by both child and parent, independently of each other.

### Concurrent validity

Adolescents who completed the PAQ-A on the same day as a cardiopulmonary exercise test (CPET) were included to study concurrent validity. These study participants were a subset of obese and normal-weight children recruited during 2012–2013 participating in a prospective study (METAFIT study) conducted at the Jessa Hospital Hasselt (Belgium). Each item of PAQ-A and the total PAQ-A score were correlated with the peak oxygen uptake, i.e. VO_2_ peak. During the CPET to volitional fatigue an electronically braked cycle (Ergofit GmbH & Co, Pirmasens, Germany) was used (cycling frequency: 70 rpm, starting and incremental load: between 10 and 40 W) [11]. These loads were based on subjects’ age, gender, height and weight, with continuous pulmonary gas exchange analysis (Jaeger Oxycon, Erich Jaeger GmbH, Germany). On the morning of each test day, a gas and volume calibration was executed. During the tests, environmental temperature was kept stable (19-21°C). Oxygen uptake (VO_2_) was collected breath-by-breath and averaged every 10 seconds. All subjects exercised to volitional exhaustion and achieved a peak respiratory gas exchange ratio (RER) >1.0.

### Physical activity questionnaire scoring

Scoring of PAQs was performed as described by Kowalski et al. [4]. PAQ-C questionnaire has been originally designed for children aged 8 to 14 and consists of nine questions structured to discern low (score 1) to high (score 5) physical activity during the last seven days and a tenth question in order to identify children or adolescents who had unusual activity during the previous week. However, the last question was not used as a part of the summary activity score.

PAQ-A questionnaire has been originally designed for adolescents aged 14 to 20. The PAQ-A questionnaire consists of only nine questions (the question about morning break was omitted, according to the original PAQ) but it has the same scoring method as PAQ-C. The first question of both PAQs contained a checklist of 22 common leisure and sport activities as well as two ‘other’ fill-in choices. The first question was scored as the mean of all activities by a score from 1 to 5. The total score of these questionnaires was calculated by adding all questions’ average scores.

### Data analysis

To determine the I-CVI, all six members of the expert committee rated each question/item of both PAQs in terms of its relevance to the underlying construct to a 4-point ordinal scale (i.e. 1: not relevant; 2: somewhat relevant; 3: quite relevant; 4: highly relevant). Consequently, the I-CVI was computed for each item as the number of experts giving a rating of either 3 or 4 divided by the total number of experts. The S-CVI was calculated for each PAQ as the average of the I-CVIs for all items on the scale. An I-CVI higher than 0.78 was assumed to be excellent, and a minimum S-CVI of 0.80 was considered as acceptable [12]. Inter-item reliability was calculated by Cronbach’s alpha (α). A value of 0.70 or higher was considered as acceptable. Inter-rater agreement was determined with kappa (κ) and 95% Confidence Intervals (CI). To permit calculation of the κ statistic, Q1, Q9 and Qtotal were transformed into quintiles before statistical analysis. Kappa values were characterized as follows: 0.00: poor agreement; 0.01-0.20: slight agreement; 0.21-0.40: fair agreement; 0.41-0.60: moderate agreement; 0.61-0.80: substantial agreement; 0.81-1.00: almost perfect agreement. Concurrent validity was assessed using Spearman correlation coefficients (r_s_) corrected for age, gender, height and weight. Data were statistically analyzed using SPSS software version IBM 20.0 (Chicago, Illinois, USA). Significance levels were set at P < 0.05.

## Results

### Content validity

Table 1 gives an overview of I-CVI and S-CVI of both PAQs based on the scoring by six professionals of the expert committee. For both PAQs, I-CVI was highest for question items 1 (i.e. sport activities in spare time), 5 (i.e. after-school activity), 6 (i.e. evening-activity) and 7 (i.e. weekend-activity), average for items 2 (i.e. activity during physical education classes) and 9 (i.e. activity during each day last week) and lowest for item 4 (i.e. lunch-time activity). Both PAQs showed a high S-CVI, i.e. 0.89 for PAQ-C and 0.90 for PAQ-A.

**Table 1:** **I-CVI and S-CVI scores for the Dutch PAQ-C and PAQ-A**

PAQ-C							
Item	Expert 1	Expert 2	Expert 3	Expert 4	Expert 5	Expert 6	I-CVI
**Q1.** Spare time activity: sports	3	4	4	4	4	4	1.00
**Q2.** Activity during physical education classes	3	3	3	3	2	3	0.83
**Q3.** Break time activity	3	3	3	2	3	2	0.67
**Q4.** Lunchtime activity	3	3	3	2	3	2	0.67
**Q5.** After-school activity	4	4	4	4	4	4	1.00
**Q6.** Evening activity	4	4	4	4	4	4	1.00
**Q7.** Weekend-activity	4	4	4	4	4	4	1.00
**Q8.** Activity frequency during the last 7 days	3	4	4	3	4	3	1.00
**Q9.** Activity frequency during each day last week	4	4	4	4	2	4	0.83
**S-CVI**							**0.89**
**PAQ-A**							
**Item**	**Expert 1**	**Expert 2**	**Expert 3**	**Expert 4**	**Expert 5**	**Expert 6**	**I-CVI**
**Q1.** Spare time activity: sports	3	4	4	4	4	4	1.00
**Q2.** Activity during physical education classes	3	3	3	3	2	3	0.83
**Q3.** Break time activity	NA	NA	NA	NA	NA	NA	NA
**Q4.** Lunchtime activity	3	3	3	3	2	2	0.67
**Q5.** After-school activity	4	4	4	4	4	4	1.00
**Q6.** Evening activity	4	4	4	4	4	4	1.00
**Q7.** Weekend-activity	4	4	4	4	4	4	1.00
**Q8.** Activity frequency during the last 7 days	2	4	4	3	4	3	0.83
**Q9.** Activity frequency during each day last week	3	4	3	4	2	4	0.83
**S-CVI**							**0.90**

I-CVI: item-level content validity; S-CVI: scale-level content validity; NA: not applicable.

### Inter-item and inter-rater reliability

The response rate of both PAQs ranged between 50 and 75%. In total, 196 PAQ-C and 95 PAQ-A were fully completed by both child and parent. Children (n = 5) who indicated they filled in the exact same answers as their parent were excluded from this analysis. Consequently, 192 PAQ-C questionnaires were completed by 101 girls and 91 boys aged between 5 and 12 (mean age: 8.9 ± 1.7 years), 94 PAQ-A questionnaires were completed by 52 girls and 42 boys aged between 12 and 17 (mean age: 13.6 ± 1.4 years) (Table 2). Adolescents scored significantly lower for Q1, Q4, Q9 (P < 0.001) and Q7 (P = 0.005) compared to children.

**Table 2:** **Descriptive characteristics of the study population**

	Inter-item and inter-rater reliability (children)		Inter-item and inter-rater reliability (parents)		Concurrent validity	Inter-item reliability (independent cohort)	
	PAQ-C (n = 192)	PAQ-A (n = 94)	PAQ-C (n = 192)	PAQ-A (n = 94)	PAQ-A (n = 44)	PAQ-C (n = 26)	PAQ-A (n = 21)
Age, years	8.9 ± 1.7^***^	13.6 ± 1.4			14.2 ± 1.8	9.1 ± 2.0^***^	13.2 ± 2.1
Gender							
Boys, n (%)	91 (47.4)	42 (44.7)	-	-	26 (59.0)	12 (46.2)	14 (66.7)
Girls, n (%)	101 (52.6)	52 (55.3)	-	-	18 (41.0)	14 (53.8)	7 (33.3)
Height, cm	138.1 ± 11.3^***^	165.0 ± 9.3	-	-	164.3 ± 10.5	138.2 ± 13.4^***^	159.2 ± 15.1
Weight, kg	32.1 ± 8.5^***^	54.6 ± 14.7	-	-	77.2 ± 26.4	38.2 ± 17.3^***^	63.6 ± 26.5
BMI, kg/m^2^	16.5 ± 2.7^***^	19.9 ± 4.1	-	-	28.2 ± 8.1	19.1 ± 5.9^**^	24.9 ± 8.5
Q1	1.50 ± 0.25^***^	1.35 ± 0.22	1.41 ± 0.21	1.30 ± 0.17	1.26 ± 0.19	1.38 ± 0.16^*^	1.27 ± 0.12
Q2	4.53 ± 0.72	4.59 ± 0.78	4.43 ± 0.76	4.50 ± 0.84	4.32 ± 1.13	4.58 ± 0.64	4.14 ± 1.11
Q3	3.94 ± 1.01	NA	3.90 ± 0.94	NA	NA	3.89 ± 0.91	NA
Q4	3.60 ± 1.21^***^	2.24 ± 1.01	3.53 ± 1.13	2.21 ± 0.97	2.34 ± 0.98	3.85 ± 1.29^***^	2.38 ± 1.16
Q5	2.49 ± 1.22	2.28 ± 1.39	2.42 ± 1.19	2.24 ± 1.33	1.95 ± 1.36	2.23 ± 1.27	2.19 ± 1.47
Q6	2.55 ± 1.09	2.38 ± 1.09	2.49 ± 1.05	2.38 ± 1.09	2.25 ± 1.22	2.39 ± 1.06	2.29 ± 1.06
Q7	2.79 ± 0.89^**^	2.46 ± 1.02	2.71 ± 0.87	2.50 ± 0.95	2.09 ± 1.20	2.50 ± 1.14	2.24 ± 1.04
Q8	2.95 ± 0.97	2.93 ± 0.96	2.87 ± 0.86	2.80 ± 0.95	2.55 ± 1.32	2.69 ± 0.97	2.76 ± 1.04
Q9	3.12 ± 0.91^***^	2.71 ± 0.81	3.01 ± 0.88	2.65 ± 0.79	2.24 ± 0.85	2.93 ± 0.82	2.46 ± 0.73
Total mean PAQ	3.05 ± 0.89^***^	2.62 ± 0.92	2.97 ± 0.89	2.57 ± 0.90	2.36 ± 0.74	2.94 ± 0.57^**^	2.47 ± 0.69

NA: not applicable. All values are presented as mean ± SD, unless otherwise indicated. Significant differences were detected between PAQ-C and PAQ-A filled in by children and adolescents using the independent samples *t* test: ^*^P < 0.05, ^**^P ≤ 0.01 and ^***^P ≤ 0.001.

The inter-item reliability, expressed by Cronbach’s α, of both PAQs was evaluated. Of all 192 PAQ-C questionnaires completed by children and all 94 PAQ-A questionnaires completed by adolescents, Cronbach’s α was 0.777 (95% CI: 0.726-0.821) and 0.758 (95% CI: 0.677-0.826), respectively.

Table 3 describes the inter-rater reliability for each question separately and for the total physical activity. For PAQ-C, percent agreement ranged 59.9-74.0% and weighted κ ranged 0.48-0.69. For PAQ-A, percent agreement ranged 51.1-77.7% and weighted κ ranged 0.51-0.68.

**Table 3:** **Inter-rater reliability for the Dutch PAQ-C and PAQ-A**

	PAQ-C (n = 192)		PAQ-A (n = 94)	
	Observed agreement (%)	Weighted κ (95% CI)	Observed agreement (%)	Weighted κ (95% CI)
**Q1.** Spare-time activity: sports	59.9	0.50 (0.41-0.60)	77.7	0.67 (0.54-0.81)
**Q2.** Activity during physical education classes	71.4	0.48 (0.37-0.59)	73.4	0.53 (0.33-0.72)
**Q3.** Break-time activity	74.0	0.64 (0.55-0.73)	NA	NA
**Q4.** Lunch-time activity	71.9	0.68 (0.60-0.77)	64.9	0.60 (0.46-0.73)
**Q5.** After-school activity	67.7	0.63 (0.54-0.71)	69.2	0.61 (0.47-0.76)
**Q6.** Evening activity	71.9	0.69 (0.62-0.77)	71.	0.68 (0.53-0.79)
**Q7.** Weekend-activity	69.8	0.56 (0.46-0.67)	57.5	0.51 (0.38-0.65)
**Q8.** Activity frequency during the last 7 days	72.4	0.65 (0.56-0.74)	70.2	0.63 (0.51-0.76)
**Q9.** Activity frequency during each day last week	65.6	0.64 (0.55-0.72)	51.0	0.51 (0.38-0.64)
**Total physical activity**	65.6	0.60 (0.52-0.67)	70.2	0.64 (0.51-0.77)

95% CI: 95% Confidence Interval; NA: not applicable.

We experienced that PAQ-C was difficult to complete for 5 and 6-year-old children (n = 24) because of their limited reading skills. Therefore, we performed an additional analysis without the 5 and 6-year-old children, which showed a good overall reliability (Cronbach’s α: 0.761; 95% CI: 0.703-0.812) and a better inter-rater reliability of total physical activity level (observed agreement of 75.0%; weighted κ: 0.72; 95% CI: 0.52-0.92) compared with the original analysis including this age group.

### Concurrent validity

A subset of 28 obese (mean BMI: 33.5 ± 4.7 kg/m^2^; mean age: 14.2 ± 1.8 years; 18 boys) and 16 normal-weight (mean BMI: 19.0 ± 2.2 kg/m^2^; mean age: 14.0 ± 1.7 years; 8 boys) adolescents completed the PAQ-A on the same day as the CPET took place. Descriptive characteristics of the study population are presented in Table 2. The mean PAQ-A score was 2.13 ± 0.68 in obese and 2.77 ± 0.68 in normal-weight individuals (P = 0.005). The total PAQ-A score was 2.36 ± 0.74. The mean VO_2_ peak was 2201 ± 500 ml/min in obese and 2498 ± 835 ml/min in normal-weight individuals (P = 0.306). The total mean VO_2_ peak was 2309 ± 650 ml/min.

PAQ-A is scored with an arbitrary numeric score, whereas the exercise monitor raw output is a scale count. The results presented in Table 4 are, therefore, representing relative validity rather than absolute validity. The associations between the PAQ-A score and VO_2_ peak were moderate to strong. Three out of eight (i.e. Q1: sport activities in spare time; Q4: lunch-time activity; Q5: after-school activity) PAQ-A items were not significantly associated with VO_2_ peak (P > 0.05). Overall, total PAQ-A score showed a moderate correlation with VO_2_ peak (r_s_ = 0.516; P = 0.001).

**Table 4:** **Concurrent validity of the PAQ-A**

	VO_2_peak
**Q1.** Spare-time activity: sports	-0.012
**Q2.** Activity during physical education classes	0.438^**^
**Q3.** Break-time activity	NA
**Q4.** Lunch-time activity	0.010
**Q5.** After-school activity	0.052
**Q6.** Evening activity	0.550^***^
**Q7.** Weekend activity	0.608^***^
**Q8.** Activity frequency during the last 7 days	0.426^**^
**Q9.** Activity frequency during each day last week	0.412^*^
**Total physical activity**	**0.516** ^**^

NA: not applicable. Spearman correlation coefficients (r_s_ corrected for age, gender, height and weight) of PAQ-A with corresponding exercise test measures of VO_2_ peak. ^*^P < 0.05; ^**^P < 0.01; ^***^P < 0.001.

### Proof of concept

We tested whether both PAQs were still reliable in a small but independent cohort consisting of 47 subjects (Table 2). These study participants were a subset of 21 overweight/obese and 26 normal-weight children recruited during 2009–2010 participating in a prospective study (COFF study [13]) conducted at the Antwerp University Hospital (Belgium). Only inter-item reliability could be calculated from both PAQs. Cronbach’s α was 0.762 (95% CI: 0.597-0.878) for PAQ-C and 0.824 (95% CI: 0.682-0.918) for PAQ-A.

## Discussion

In this study, we evaluated the reliability and validity of a Dutch version of the PAQ-C and PAQ-A in a convenient sample of children and adolescents living in Flanders, Belgium. We report excellent content validity, an acceptable inter-item reliability and moderate to good strength of inter-rater agreement of PAQ-C (completed by children aged 5 to 12) and PAQ-A (completed by adolescents aged 12 to 17). In addition, the concurrent validity correlation of PAQ-A with the VO_2_ peak was moderate.

To describe the level and pattern of physical activity, a standardized, reliable and valid instrument is essential. Furthermore, in children and adolescents it is important to use instruments which are non-invasive, easy-to-use and time-saving. To our knowledge, there are only a few validated short questionnaires in Dutch to assess the overall level of physical activity in adolescents [2, 14] and the usefulness of these questionnaires in children younger than 11 years has not yet been assessed. A thorough literature study revealed that the original Canadian PAQ [4] – a short standardized questionnaire – was suited for the evaluation of the overall physical activity level in both children (PAQ-C) and adolescents (PAQ-A). Moreover, in a recent review by Biddle et al. [15], different self-reported physical activity instruments developed for use in children and adolescents were compared in order to assess their suitability and feasibility for the use in population surveillance systems and tracking trends over time. In total, they identified 20 activity-based instruments of which three were supported by the majority of the expert group and authors, namely PAQ-C and PAQ-A, Youth Risk Behaviour Surveillance Survey (YRBS) and the Teen Health Survey. These physical activity measurement instruments demonstrated not only reliability and validity but also ease of use. In addition, it was noted that no data are currently available on the use of both PAQs in Europe [15]. Therefore, we evaluated the reliability and validity of a Dutch version of both PAQ-C and PAQ-A in a convenient sample of children and adolescents living in Flanders.

In our study, adolescents generally showed lower levels of physical activity compared to children. Janz et al. [8] also found a lower level of physical activity in adolescents (PAQ-A score: 2.51 ± 0.61) compared to children (PAQ-C score: 2.61 ± 0.60), although this was not tested for significance. The lower physical activity observed during adolescence might be explained by a decrease in non-organized sport and vigorous physical activity [16]. This explanation fits well in the further interpretation of our results, since we observed higher scores of PAQ-A for the second question. This question handles about the frequency of being active during the physical education classes. Therefore, we assume that adolescents are more active during organized sport activities, but fail in maintaining a high level of physical activity during playtimes and activities outside school. In addition, the low values of PAQ-A observed in our study samples of concurrent validity and inter-item reliability tested in the independent cohort, were due to the characteristics of the study populations containing obese children and adolescents.

Content validity of the Dutch PAQ-C and PAQ-A was evaluated by the expert committee and was found to be excellent. Questions concerning sport activities in spare time, after-school, in the evening and the weekend were found highly relevant, whereas questions concerning break and lunchtime were scored lowest. This finding can be explained by the fact that break and lunchtime activities at school are relatively short in time and depend on whether the school itself organizes sport activities during break or lunch. Sport activities in spare time, after-school, in the evening or weekend are often practiced in sports associations and last longer in time. Nonetheless, we can conclude that the content of both PAQs is very relevant.

PAQ-C and PAQ-A showed an acceptable inter-item reliability, which is in line with previous reports [5, 8, 17, 18]. As a proof of concept, we re-tested inter-item reliability in a small but independent cohort and still found a good internal consistency for both PAQs.

Because of the short time frame of our study and from practical point of view we opted to study inter-rater instead of test-retest reliability. We found a moderate to good strength of inter-rater agreement. Accordingly, we conclude that these questionnaires are reliable for their use in the assessment of the overall level of physical activity in children and adolescents speaking Dutch. However, some marginal notes have to be made. Firstly, both PAQs can also be implemented in other Dutch speaking countries provided that the sport activities practiced in the respective country are used in the questionnaires. Nevertheless, caution has to be taken since Moore et al. [18] proved that reliability and validity of the PAQ-C differed between ethnic populations, i.e. African American, European American and Hispanic children. Moreover, the sport activities should always be reconsidered in time according to the existing sport trends. Also important to note is the high level of parent–child agreement for PAQ-C completed by 5 and 6-year-old children. This is probably due to the fact that parents helped their children with completing the questionnaire, but did not report this to the investigators. It is therefore important to inform the parents that they may help the child with reading and completing the questions, but that they should not provide any guidance in answering the questions.

The concurrent validity correlation between the Dutch PAQ-A total score and the VO_2_ peak was moderate. However, it is considerable higher than the previously reported associations between PAQ-A and accelerometry [5, 8, 17]. Consequently, PAQ-A might serve as a valid tool to assess physical activity level in adolescents.

## Conclusions

This is the first validation study of a Dutch version of the PAQ-C and PAQ-A questionnaires for children and adolescents. Our results show that the Dutch versions of both PAQ-C and PAQ-A, provide reliable and valid estimates of physical activity among 5 to 17-year-old children and adolescents. Both questionnaires can be considered as very useful in clinical practice to assess overall level of physical activity in children and adolescents. Eventually, assessment of physical activity both at individual and at population level, could lead to improvement of personalized interventions and new school policies in order to prevent as well as to combat weight gain and associated health complications.

## Electronic supplementary material

Additional file 1: Dutch Physical Activity Questionnaire for Children (PAQ-C).(PDF 175 KB)

Additional file 2: Dutch Physical Activity Questionnaire for Adolescents (PAQ-A).(PDF 118 KB)
