# Supplementary material for: Reliability and Validity of the Dutch Physical Activity Questionnaires for Children (PAQ-C) and Adolescents (PAQ-A)
Source: Arch Public Health. 2014 Dec 24;72:47. doi: 10.1186/2049-3258-72-47 (PMC4323128; doi:10.1186/2049-3258-72-47)
Supplement: Supplementary file 1 — Additional file 1: Dutch Physical Activity Questionnaire for Children (PAQ-C).(PDF 175 KB) [file 13690_2014_5057_MOESM1_ESM.pdf]

Additional file 1

Dutch Physical Activity Questionnaire for Children (PAQ-C)

Vragenlijst fysieke activiteit (lagere school)

Naam:.. Leeftijd:...................................

Geslacht: Meisje / Jongen Leerjaar:...................................

We willen met deze vragenlijst een beeld krijgen over het niveau van jouw fysieke activiteiten gedurende **de voorbije 7 dagen** (dus de voorbije week). Met deze activiteiten bedoelen we sporten of dansen waarvan je gaat zweten of waarbij je benen moe aanvoelen, of spelletjes waardoor je sneller gaat ademen, zoals tikkertje, touwtje springen, rennen, klimmen en andere.

Onthoud het volgende:

1. Dit is geen test! Er zijn dus geen juiste of foute antwoorden.
2. Probeer alsjeblieft de vragen zo eerlijk en correct mogelijk te beantwoorden. Dit is voor ons heel belangrijk.

---

1. Fysieke activiteit in je vrije tijd: heb je één of meer van de volgende activiteiten beoefend in de voorbije 7 dagen (afgelopen week)? Zo ja, hoeveel keer? (Slechts 1 bolletje per rij aankleuren)

	Niet	1-2	3-4	5-6	7 keer of meer
Touwspringen	O	O	O	O	O
Tennis	O	O	O	O	O
In-line skating	O	O	O	O	O
Tikkertje spelen	O	O	O	O	O
Wandelen als sport	O	O	O	O	O
Fietsen	O	O	O	O	O
Joggen of rennen	O	O	O	O	O
Atletiek	O	O	O	O	O
Zwemmen	O	O	O	O	O
Baseball, honkbal	O	O	O	O	O
Dansen	O	O	O	O	O
Rugby	O	O	O	O	O
Badminton	O	O	O	O	O
Skateboarden	O	O	O	O	O
Voetbal	O	O	O	O	O
Hockey	O	O	O	O	O
Volleybal	O	O	O	O	O

Gevechtssporten	O	O	O	O	O
Basketbal	O	O	O	O	O
IJsschaatsen	O	O	O	O	O
Paardrijden	O	O	O	O	O
Turnen	O	O	O	O	O
Andere:					
....	O	O	O	O	O
....	O	O	O	O	O

2. Hoe vaak ben je de voorbije 7 dagen erg actief geweest tijdens de turnlessen? (Hiermee bedoelen we rennen, hevig spelen, springen, gooien) (Slechts 1 bolletje aankleuren)

   Ik doe niet mee tijdens de turnlessen............ O
   Bijna nooit............ O
   Soms............ O
   Bijna altijd............ O
   Altijd............ O

3. Wat heb je de voorbij 7 dagen het grootste deel van de tijd gedaan *tijdens de speeltijd* op school? (Slechts 1 bolletje aankleuren)

   Zitten (praten, lezen, huiswerk maken)............ O
   Rechtstaan of rondwandelen............ O
   Een beetje rondlopen of spelen............ O
   Redelijk veel rondlopen of spelen .... O
   Bijna steeds rondlopen of hevig spelen............ O

4. Wat heb je de voorbije 7 dagen meestal gedaan *gedurende de middagpauze* (behalve het eten van je middagmaal)? (Slechts 1 bolletje aankleuren)

   Zitten (praten, lezen, huiswerk maken)............ O
   Rechtstaan of rondwandelen............ O
   Een beetje rondlopen of spelen............ O
   Redelijk veel rondlopen .... O
   Bijna steeds rondlopen of hevig spelen............ O

5. Hoeveel keer in de voorbije 7 dagen heb je *onmiddellijk na school* gesport, gedanst of een spel gespeeld waarbij je heel actief was? (Slechts 1 bolletje aankleuren)

Geen enkele keer..O
1 keer de afgelopen week...O
2 of 3 keer afgelopen week..O
4 keer de afgelopen week ....O
5 keer de afgelopen week...O

6. Hoeveel keer in de voorbije 7 dagen heb je *'s avonds* gesport, gedanst of een spel gespeeld waarbij je heel actief was? (Slechts 1 bolletje aankleuren)

Geen enkele keer..O
1 keer de afgelopen week...O
2 of 3 keer afgelopen week..O
4 keer de afgelopen week ....O
5 keer de afgelopen week...O

7. Hoeveel keer heb je *in het voorbije weekend* gesport, gedanst of een spel gespeeld waarbij je heel actief was? (Slechts 1 bolletje aankleuren)

Geen enkele keer..O
1 keer de afgelopen week...O
2 of 3 keer afgelopen week..O
4 keer de afgelopen week ....O
5 keer de afgelopen week...O

8. Welke van de volgende stellingen beschrijft jou hebt beste in de voorbij 7 dagen? Lees eerst de 5 stellingen alvorens je één antwoord kiest dat het best bij je past. (Omcirkel de letter van één stelling)

A. Zelden of nooit heb ik in mijn vrije tijd fysieke activiteiten gedaan zoals sporten, lopen, zwemmen, fietsen, aerobics, ...
B. Soms (1-2 keer de voorbije week) heb ik fysieke activiteiten gedaan in mijn vrije tijd (zoals een bepaalde sport beoefend, gaan lopen, zwemmen, fietsen, aerobics)
C. Vaak (3-4 keer de voorbije week) heb ik fysieke activiteiten gedaan in mijn vrije tijd
D. Erg vaak (5-6 keer de voorbije week) heb ik fysieke activiteiten gedaan in mijn vrije tijd
E. Zeer vaak (7 keer of meer de voorbije week) heb ik fysieke activiteiten gedaan in mijn vrije tijd

9. Duidt aan hoe vaak je actief was (zoals een sport beoefenen, spelen, dansen of elke andere fysieke activiteit) voor elke dag van de voorbije week.

	Niet	Een beetje	Gemiddeld	Vaak	Bijna altijd
Maandag	O	O	O	O	O
Dinsdag	O	O	O	O	O
Woensdag	O	O	O	O	O
Donderdag	O	O	O	O	O
Vrijdag	O	O	O	O	O
Zaterdag	O	O	O	O	O
Zondag	O	O	O	O	O

10. Was je de voorbije week ziek of was er iets waardoor je je normale fysieke activiteiten niet kon uitvoeren? (Slechts 1 bolletje aankleuren)

Ja..O
Nee..O

Zo ja, wat was de oorzaak? ....
