# Supplementary material for: Reliability and Validity of the Dutch Physical Activity Questionnaires for Children (PAQ-C) and Adolescents (PAQ-A)
Source: Arch Public Health. 2014 Dec 24;72:47. doi: 10.1186/2049-3258-72-47 (PMC4323128; doi:10.1186/2049-3258-72-47)
Supplement: Supplementary file 2 — Additional file 2: Dutch Physical Activity Questionnaire for Adolescents (PAQ-A).(PDF 118 KB) [file 13690_2014_5057_MOESM2_ESM.pdf]

Additional file 2

Dutch Physical Activity Questionnaire for Adolescents (PAQ-A)

Vragenlijst Fysieke Activiteit (Middelbare School)

Naam:... Leeftijd:.....................................

Geslacht: M/V Studiejaar:.................................

Met deze vragenlijst willen we een beeld krijgen van het niveau van jouw fysieke activiteiten van **de voorbije 7 dagen** (dus de voorbije week). Met deze activiteiten bedoelen we sporten of dansen waarvan je gaat zweten of waarbij je benen moe aanvoelen of spelletjes waardoor je sneller gaat ademen, zoals tikkertje, touwtjespringen, rennen, klimmen en andere.

Onthoud het volgende:

1. Dit is geen test! Er zijn dus geen juiste of foute antwoorden.
2. Gelieve de vragen zo eerlijk en correct mogelijk in te vullen. Dit is voor ons zeer belangrijk.

---

1. Fysieke activiteit in je vrije tijd: Heb je één of meer van de volgende activiteiten in de voorbije 7 dagen (voorbije week) beoefend? Zo ja, hoeveel keer? (Kleur per rij slechts 1 bolletje in)

	Niet	1-2	3-4	5-6	of meer
Touwspringen	O	O	O	O	O
Tennis	O	O	O	O	O
In-line skating	O	O	O	O	O
Tikkertje spelen	O	O	O	O	O
Wandelen als sport	O	O	O	O	O
Fietsen	O	O	O	O	O
Lopen of joggen	O	O	O	O	O
Atletiek	O	O	O	O	O
Zwemmen	O	O	O	O	O
Baseball, honkbal	O	O	O	O	O
Dansen	O	O	O	O	O
Rugby	O	O	O	O	O
Badminton	O	O	O	O	O
Skateboarden	O	O	O	O	O
Voetbal	O	O	O	O	O
Hockey	O	O	O	O	O
Volleybal	O	O	O	O	O
Gevechtssporten	O	O	O	O	O
Basketbal	O	O	O	O	O

Ijsschaatsen	O	O	O	O	O
Turnen	O	O	O	O	O
Paardrijden	O	O	O	O	O
Andere:					
………………	O	O	O	O	O
………………	O	O	O	O	O

2. Hoe vaak ben je de voorbije 7 dagen erg actief geweest tijdens de turnlessen (actief spelen, lopen, springen, werpen)? (Kleur slechts één bolletje)

Ik doe niet mee tijdens de turnlessen……………………….………. O
Bijna nooit………………………………………………………… O
Soms……………………………………………………………… O
Meestal…………………………………………………………. O
Altijd……………………………………………………………… O

3. Wat heb je in de voorbije 7 dagen meestal gedaan tijdens de lunchpauze, behalve het eten van je middagmaal? (Kleur slechts één bolletje)

Zitten (praten,lezen,huiswerk,…)………………………………. O
Rechtstaan of rondwandelen……………………………………. O
Beetje rondlopen of spelen……………………………………… O
Veel rondlopen of veel spelen……………………………….. O
Zeer veel rondlopen of zeer veel spelen…………………………. O

4. Hoeveel keer in de voorbije 7 dagen heb je, *onmiddellijk na school*, gesport, gedanst of een spel gespeeld waarbij je zeer actief was? (Kleur slechts één bolletje)

Niet………………………………………………………….…….…O
1 keer in de voorbije week…………………………………………..O
2 of 3 keer in de voorbije week……………………………………..O
4 keer in de voorbije week……………………………………………O
5 keer in de voorbije week …………………...………………………..O

5. Hoeveel keer in de voorbije 7 dagen heb je '*s avonds* gesport, gedanst of een spel gespeeld waarbij je zeer actief was? (Kleur slechts één bolletje)

Niet…………………………………………………………….…….…O
1 keer in de voorbije week……………………………………………..O
2 of 3 keer in de voorbije week……………………….………………..O
4 of 5 keer in de voorbije week……………….…………….……………O
6 of 7 keer in de voorbije week ………………………………………..O

6. Hoeveel keer heb je *in het voorbije weekend* gesport, gedanst of een spel gespeeld waarbij je zeer actief was? (Kleur slechts één bolletje)

Niet……………………………………………………………….……..…	O
1 keer ……………………….……….……………………………….	O
2 - 3 keer…..…………………….…..………………………………	O
4 - 5 keer………………………..……………………………….	O
6 keer of meer…………………..……....…………………………………..	O

7. Welk van de volgende stellingen beschrijft jou het best voor de voorbije 7 dagen? Lees eerst alle 5 de stellingen alvorens je één antwoord kiest dat het beste bij je past. (Omcirkel de letter van één stelling)

A. Zelden of nooit heb ik in mijn vrije tijd fysieke activiteiten gedaan (vb. sporten, lopen, zwemmen, fietsen, aerobics,…)

B. Soms (1 – 2 keer in de voorbije week) heb ik fysieke activiteiten gedaan in mijn vrije tijd (vb. sporten, lopen, zwemmen, fietsen, aerobics, ….)

C. Frequent (3 – 4 keer in de voorbije week) heb ik fysieke activiteiten gedaan in mijn vrije tijd

D. Heel frequent (5 – 6 keer in de voorbije week)heb ik fysieke activiteiten gedaan in mijn vrije tijd

E. Zeer frequent (7 keer of meer in de voorbije week) heb ik fysieke activiteiten gedaan in mijn vrije tijd

8. Duidt aan hoe vaak je aan fysieke activiteit deed (zoals sporten, spelen, dansen of iets anders) voor elke dag van de voorbije week.

	Niet	Een beetje	Medium	Vaak	Bijna altijd
Maandag……………	O	O	O	O	O
Dinsdag……………	O	O	O	O	O
Woensdag……………	O	O	O	O	O
Donderdag……………	O	O	O	O	O
Vrijdag……………	O	O	O	O	O
Zaterdag……………	O	O	O	O	O
Zondag……………	O	O	O	O	O

9. Ben je de voorbije week ziek geweest of heb je iets speciaals gedaan waardoor je jouw normale fysieke activiteiten niet kon doen?

Ja…………………………………………………………………..…... O
Neen …………………………………………………………………... O

Indien ja, wat was de oorzaak?...
